# Supplementary material for: Association of OCT biomarkers and visual impairment in patients with diabetic macular oedema with vitreomacular adhesion
Source: PLoS One. 2023 Jul 18;18(7):e0288879. doi: 10.1371/journal.pone.0288879 (PMC10353796; doi:10.1371/journal.pone.0288879)
Supplement: Attachment — Submitted filename: PLOS-ONE OCT biomarkers.docx [file pone.0288879.s002.docx]

Abbreviations like HRD, DRILL , although mentioned and explained later in the test , they need to be clarified earlier in the abstract.

**Line 12 :** inner layers cysts need more clarification ( Inner Nuclear Layer INL , Inner plexiform Layers IPL or Ganglion Cell Layer GCC )

**Line 13 :** bridging tissue , needed earlier explanation , Muller cell fibers , Muller cell Pillars

**Line 98**: DRIL definition needs revision Disorganization of Retinal Inner Layers , NOT Disruption and this applies to the whole text . The original definition of DRIL also included disorganization of Retinal inner layers in central 1000 microns . The need revision in the test and also in the methodology

**Line 137 :** difference in the age …. Needs more clarification which group older or younger age

**Line 138 :** need adding units (years ) and explanation ( mean , range )

**Line 140 / 148 :** results need more clarification of the different groups

**Table 2 :** DRIL Disorganization NOR disruption of Retinal Inner layers

**Line 198 :** VMA by definition NOT causing any traction . If there is antero posterior or tangential traction , it is then Vitreo macular Traction ( VMT) and not VMA. Inclusion criteria mentioned VMA and not VMT .

**Line 202 :** Sun et al missing year , this applied to all other references

**Line 204 :** Fovea involving DME , the correct term Center – involving DME

In discussion more explanation needed to explain how VMA WITHOUT VMT can affect vision
