# Supplementary material for: Association of OCT biomarkers and visual impairment in patients with diabetic macular oedema with vitreomacular adhesion
Source: PLoS One. 2023 Jul 18;18(7):e0288879. doi: 10.1371/journal.pone.0288879 (PMC10353796; doi:10.1371/journal.pone.0288879)
Supplement: Attachment — Submitted filename: Response to Reviewers.docx [file pone.0288879.s003.docx]

**Rebuttal letter PLOS One**

Journal Requirements:

When submitting your revision, we need you to address these additional requirements.

1. Please ensure that your manuscript meets PLOS ONE's style requirements, including those for file naming. The PLOS ONE style templates can be found at

<https://journals.plos.org/plosone/s/file?id=wjVg/PLOSOne_formatting_sample_main_body.pdf> and

<https://journals.plos.org/plosone/s/file?id=ba62/PLOSOne_formatting_sample_title_authors_affiliations.pdf>

Response: Manuscript edited according to journal style

2. Thank you for stating the following financial disclosure:

“none”

At this time, please address the following queries:

a) Please clarify the sources of funding (financial or material support) for your study. List the grants or organizations that supported your study, including funding received from your institution.

b) State what role the funders took in the study. If the funders had no role in your study, please state: “The funders had no role in study design, data collection and analysis, decision to publish, or preparation of the manuscript.”

c) If any authors received a salary from any of your funders, please state which authors and which funders.

d) If you did not receive any funding for this study, please state: “The authors received no specific funding for this work.”

Please include your amended statements within your cover letter; we will change the online submission form on your behalf.

Response: Statement mentioned in the cover letter- as The authors received no specific funding for this work.”

3. Thank you for stating the following in your Competing Interests section:

“none”

Please complete your Competing Interests on the online submission form to state any Competing Interests. If you have no competing interests, please state "The authors have declared that no competing interests exist.", as detailed online in our guide for authors at <http://journals.plos.org/plosone/s/submit-now>

 This information should be included in your cover letter; we will change the online submission form on your behalf.

Response: Statement mentioned in the cover letter - The authors have declared that no competing interests exist.

4. We note that you have indicated that data from this study are available upon request. PLOS only allows data to be available upon request if there are legal or ethical restrictions on sharing data publicly. For more information on unacceptable data access restrictions, please see <http://journals.plos.org/plosone/s/data-availability#loc-unacceptable-data-access-restrictions>.

In your revised cover letter, please address the following prompts:

a) If there are ethical or legal restrictions on sharing a de-identified data set, please explain them in detail (e.g., data contain potentially sensitive information, data are owned by a third-party organization, etc.) and who has imposed them (e.g., an ethics committee). Please also provide contact information for a data access committee, ethics committee, or other institutional body to which data requests may be sent.

b) If there are no restrictions, please upload the minimal anonymized data set necessary to replicate your study findings as either Supporting Information files or to a stable, public repository and provide us with the relevant URLs, DOIs, or accession numbers. For a list of acceptable repositories, please see <http://journals.plos.org/plosone/s/data-availability#loc-recommended-repositories>.

We will update your Data Availability statement on your behalf to reflect the information you provide.

Response: There are no restrictions in sharing the data and excel data supporting our study is provided as supporting information files as S1 Data (XLS)

5. Please include your full ethics statement in the ‘Methods’ section of your manuscript file. In your statement, please include the full name of the IRB or ethics committee who approved or waived your study, as well as whether or not you obtained informed written or verbal consent. If consent was waived for your study, please include this information in your statement as well.

Response: The name of the IRB is Vision Research Foundation and changes are mentioned at line 68

6. Please review your reference list to ensure that it is complete and correct. If you have cited papers that have been retracted, please include the rationale for doing so in the manuscript text, or remove these references and replace them with relevant current references. Any changes to the reference list should be mentioned in the rebuttal letter that accompanies your revised manuscript. If you need to cite a retracted article, indicate the article’s retracted status in the References list and also include a citation and full reference for the retraction notice.

Response: References reviewed there are no retracted articles cited in this manuscript. The reference list has been modified to incorporate additional articles during the revision process.

Additional Editor Comments:

Thank you for submitting this article 
Minor Revision needed

Abbreviations like HRD, DRILL, although mentioned and explained later in the test , they need to be clarified earlier in the abstract.

Response: Abbreviations are clarified

**Line 12  :** inner layers cysts need more clarification ( Inner Nuclear Layer INL , Inner plexiform Layers IPL or Ganglion Cell Layer GCC )

Response: Clarification given

**Line 13 :** bridging tissue , needed earlier explanation , Muller cell fibers , Muller cell Pillars

Response: Explanation given

**Line 98**:  DRIL definition needs revision Disorganization of Retinal Inner Layers , NOT Disruption  and this applies to the whole text . The original definition of DRIL also included disorganization of Retinal inner layers in central 1000 microns . The need revision in the test and also in the methodology

Response: Revision done and changes are mentioned in methodology.

**Line 137 :** difference in the age …. Needs more clarification which group older or younger age

Response: Clarification given

Response: Table 1 presents the demographic and baseline characteristics of the participants in the study. The three groups, namely focal VMA, broad VMA, and the control group, exhibited similar distributions in terms of gender, duration of diabetes (focal VMA: 11years [8-20], broad VMA: 15 years [10-18] and control: 12 years [8-17]), presence of other systemic diseases, retinopathy grades, lens status, previous treatments, mean number of injections (focal VMA: 2 injections [1-3], broad VMA: 2 injections [1-3] and control: 2 injections [1-3]) and distance vision (focal VMA: 0.50 [0.20-0.70], broad VMA: 0.40 [0.20-0.60] and control: 0.30 [0.20-0.60]). However, there was a significant difference in observed in age (p=0.020) among the three groups, focal VMA group had a higher median age of 63 years (57-67) years, compared to the broad VMA group 60 years (53-65), and control group 59 years (51-64). Additionally, the values of CRT (p<0.001) and CSFT (p<0.001) also displayed significant difference among the three groups (focal VMA, broad VMA and the control group).

**Line 138:** need adding units (years) and explanation (mean, range)

Response: Clarification given

Response: Table 1 presents the demographic and baseline characteristics of the participants in the study. The three groups, namely focal VMA, broad VMA, and the control group, exhibited similar distributions in terms of gender, duration of diabetes (focal VMA: 11years [8-20], broad VMA: 15 years [10-18] and control: 12 years [8-17]), presence of other systemic diseases, retinopathy grades, lens status, previous treatments, mean number of injections (focal VMA: 2 injections [1-3], broad VMA: 2 injections [1-3] and control: 2 injections [1-3]) and distance vision (focal VMA: 0.50 [0.20-0.70], broad VMA: 0.40 [0.20-0.60] and control: 0.30 [0.20-0.60]). However, there was a significant difference in observed in age (p=0.020) among the three groups, focal VMA group had a higher median age of 63 years (57-67) years, compared to the broad VMA group 60 years (53-65), and control group 59 years (51-64). Additionally, the values of CRT (p<0.001) and CSFT (p<0.001) also displayed significant difference among the three groups (focal VMA, broad VMA and the control group).

**Line 140 / 148:** results need more clarification of the different groups

Response: Clarification given

Response: Table 1 presents the demographic and baseline characteristics of the participants in the study. The three groups, namely focal VMA, broad VMA, and the control group, exhibited similar distributions in terms of gender, duration of diabetes (focal VMA: 11years [8-20], broad VMA: 15 years [10-18] and control: 12 years [8-17]), presence of other systemic diseases, retinopathy grades, lens status, previous treatments, mean number of injections (focal VMA: 2 injections [1-3], broad VMA: 2 injections [1-3] and control: 2 injections [1-3]) and distance vision (focal VMA: 0.50 [0.20-0.70], broad VMA: 0.40 [0.20-0.60] and control: 0.30 [0.20-0.60]). However, there was a significant difference in observed in age (p=0.020) among the three groups, focal VMA group had a higher median age of 63 years (57-67) years, compared to the broad VMA group 60 years (53-65), and control group 59 years (51-64). Additionally, the values of CRT (p<0.001) and CSFT (p<0.001) also displayed significant difference among the three groups (focal VMA, broad VMA and the control group).

**Table 2  :** DRIL  Disorganization NOR disruption of Retinal Inner layers

Response: Changes are done wherever required

**Line 198 :** VMA by definition NOT causing any traction . If there is antero posterior or tangential traction , it is then Vitreo macular Traction ( VMT) and not VMA. Inclusion criteria mentioned VMA and not VMT .

Response: Changes mentioned in line 243-248

We noticed that the CRT and CSFT values were higher among the broad VMA group when compared to the focal VMA and control groups. This is an interesting finding. VMA is generally thought to not contribute much to the retinal thickening, but the retinal thickening in VMA associated with DME is assumed to be caused due to the up regulation of VEGF and vascular hyper permeability. ^[11]^

**Line 202 :** Sun et al missing year , this applied to all other references

Response: Changes are done in line 250

**Line 204 :** Fovea involving DME  , the correct term Center – involving DME

Response: Changes are mentioned in line 252

In discussion  more explanation needed to explain how VMA WITHOUT VMT can affect vision

Response: Clarification given in line 272-283

VMA can be associated with various known retinal diseases that cause visual dysfunction. ^[6,33-40]^ Several studies provide evidence supporting this hypothesis, suggesting that the occurrence of specific retinal complications is significantly higher in eyes with VMA compared to those with vitreomacular separation. ^[6,33-40]^ This finding aligns with the widely acceptable understanding that VMA eyes exhibit stronger and persistent adhesions at fovea, resulting in detrimental complications. ^[6,33-40]^ Our study findings were also consistent with the previous literature. We observed a similar prevalence of biomarkers in eyes with or without VMA, but the presence of these biomarkers was more likely to be associated with visual impairment in eyes with VMA. Additionally, in the presence of VMA, the biomarkers appeared to have a greater extent and severity, which predisposed the eyes to a poorer visual prognosis. Hence, VMA can be considered an additional biomarker indicative of a poor visual prognosis.

Therefore, it is crucial to emphasize accurate diagnosis, long-term monitoring, and appropriate management when dealing with patients who experience adverse consequences associated with vitreomacular adhesion (VMA). In this regard, spectral domain optical coherence tomography (SD-OCT) emerges as a clinically valuable and readily available method that can identify specific abnormalities at the vitreomacular interface that might not be detectable using ultrasonography or indirect ophthalmoscopy. ^[6,33,37,41]^

Reviewers' comments:

Reviewer's Responses to Questions

**Comments to the Author**

1. Is the manuscript technically sound, and do the data support the conclusions?

The manuscript must describe a technically sound piece of scientific research with data that supports the conclusions. Experiments must have been conducted rigorously, with appropriate controls, replication, and sample sizes. The conclusions must be drawn appropriately based on the data presented.

Reviewer #1: Yes

Response: Thank you for the response

2. Has the statistical analysis been performed appropriately and rigorously?

Reviewer #1: I Don't Know

3. Have the authors made all data underlying the findings in their manuscript fully available?

The [PLOS Data policy](http://www.plosone.org/static/policies.action#sharing) requires authors to make all data underlying the findings described in their manuscript fully available without restriction, with rare exception (please refer to the Data Availability Statement in the manuscript PDF file). The data should be provided as part of the manuscript or its supporting information, or deposited to a public repository. For example, in addition to summary statistics, the data points behind means, medians and variance measures should be available. If there are restrictions on publicly sharing data—e.g. participant privacy or use of data from a third party—those must be specified.

Reviewer #1: Yes

Response: Thank you for the response

4. Is the manuscript presented in an intelligible fashion and written in standard English?

PLOS ONE does not copyedit accepted manuscripts, so the language in submitted articles must be clear, correct, and unambiguous. Any typographical or grammatical errors should be corrected at revision, so please note any specific errors here.

Reviewer #1: Yes

Response: Thank you for the response

5. Review Comments to the Author

Please use the space provided to explain your answers to the questions above. You may also include additional comments for the author, including concerns about dual publication, research ethics, or publication ethics. (Please upload your review as an attachment if it exceeds 20,000 characters)

Reviewer #1: An interesting retrospective study with some new observations.
Explain the following abbreviations in the abstract : HR dots and DRIL.
Kindly review lines 210-212.
Add recommendations at the end.

Response: Thank you for the response, the explanation for HR Dots and DRILL is mentioned in abstract

Revision for the lines 210-212 is as follows:

Al Fran et al (2014), it was found that the presence of bridging tissue between cystic cavities is linked to improved functional outcomes after receiving bevacizumab injections. The absence of the bridging process, on the other hand, is associated with poorer outcomes, and leading to retinal thinning and atrophy. ^[26]^ In our current study, we observed higher percentage of eyes (76.92%) within the broad VMA group that lacked bridging lines. These findings align with the previous literature and suggest that the outcomes for the broad VMA group are likely to be unfavourable, as indicated by the absence of bridging tissue. 9These changes are done in line 256-263)

Recommendations:

As mentioned earlier, VMA may contribute to the development of various retinal complications. To ensure early detection and effective management of subsequent visual complications, it is advisable to closely monitor VMA in the long term. Future studies can be conducted prospectively to analyse the progression of VMA, particularly in terms of identifying any traction leading to macular holes. When general ophthalmologists encounter patients with VMA, it is recommended to schedule regular follow-ups, especially utilizing tests like monthly SD-OCT, if available, to specifically evaluate the vitreomacular interface. If any significant visual dysfunction or retinal complications are identified, it is important to promptly refer the patient to a vitreoretinal specialist.

6. PLOS authors have the option to publish the peer review history of their article ([what does this mean?](https://journals.plos.org/plosone/s/editorial-and-peer-review-process#loc-peer-review-history)). If published, this will include your full peer review and any attached files.


If you choose “no”, your identity will remain anonymous but your review may still be made public.


**Do you want your identity to be public for this peer review?** For information about this choice, including consent withdrawal, please see our [Privacy Policy](https://www.plos.org/privacy-policy).

Reviewer #1: No
